# High-Throughput Sequencing Approach to Analyze the Effect of Aging Time and Barrel Usage on the Microbial Community Composition of Red Wines

**DOI:** 10.3389/fmicb.2020.562560

**Published:** 2020-09-09

**Authors:** Dimitrios Kioroglou, Albert Mas, Maria C. Portillo

**Affiliations:** Department Bioquímica i Biotecnologia, Facultat d‘Enologia, Universitat Rovira i Virgili, Tarragona, Spain

**Keywords:** aged wine, oak barrels, high-throughput sequencing, bacterial communities, fungal communities

## Abstract

Wine aged in barrels or bottles is susceptible to alteration by microorganisms that affect the final product quality. However, our knowledge of the microbiota during aging and the factors modulating the microbial communities is still quite limited. The present work uses high-throughput sequencing (HTS) techniques to deal with the meta-taxonomic characterization of microbial consortia present in red wines along 12 months aging. The wines obtained from two different grape varieties were aged at two different cellars and compared based on time of wine aging in the barrels, previous usage of the barrels, and differences between wine aging in oak barrels or glass bottles. The aging in barrels did not significantly affect the microbial diversity but changed the structure and composition of fungal and bacterial populations. The main microorganisms driving these changes were the bacterial genera *Acetobacter*, *Oenococcus*, *Lactobacillus*, *Gluconobacter*, *Lactococcus*, and *Komagataeibacter* and the fungal genera *Malassezia*, *Hanseniaspora*, and *Torulaspora*. Our results showed that the oak barrels increased effect on the microbial diversity in comparison with the glass bottles, in which the microbial community was very similar to that of the wine introduced in the barrels at the beginning of the aging. Furthermore, wine in the bottles harbored higher proportion of *Lactobacillus* but lower proportion of *Acetobacter*. Finally, it seems that 1 year of previous usage of the barrels was not enough to induce significant changes in the diversity or composition of microbiota through aging compared with new barrels. This is the first meta-taxonomic study on microbial communities during wine aging and shows that the microorganism composition of barrel-aged wines was similar at both cellars. These results hint at the possibility of a common and stable microbiota after aging in the absence of exogenous alterations. Further corroborations on the current outcome would be valuable for the comparison and detection of microbial alterations during aging that could potentially prevent economic losses in the wine industry.

## Introduction

Winemaking is a process in which *Saccharomyces cerevisiae* is the main yeast responsible for the alcoholic fermentation of grape must to produce wine. However, a wide diversity of yeast and bacterial species from the grape surfaces, the field, or the cellar facilities and equipment might contribute to the final wine quality (Ribéreau-Gayon et al., 2006; Bokulich et al., 2016). These microorganisms can exert a positive or negative influence through all the winemaking process including wine maturation and aging (Renouf et al., 2005; Moreno-Arribas and Carmen Polo, 2008). The maturation and aging process starts with the introduction of wine in wooden barrels, and it continues after bottling until its consumption. Nowadays, the wood barrel aging is a common practice in winemaking for wine maturation of higher red quality wine (Ortega-Heras et al., 2007). The main reason is that barrel aging improves many red wines from a visual, olfactory, and gustatory point of view because the extractable compounds of the casks induce positive changes in the composition and flavor of the aged wine (Spillman et al., 1997; Garde-Cerdán and Ancín-Azpilicueta, 2006; Cadahía et al., 2009; Gómez-Plaza and Cano-López, 2011). However, during aging, microorganisms surviving the winemaking process remain in the pores of the wood or occasional contaminants might produce metabolic compounds that can cause deviations from the olfactory optimum and spoilage of the wine. Bottle aging is also susceptible to undesirable microbial growth even though fining or racking (filtration and clarification) are applied with the aim of microbiological stabilization (Du Toit et al., 2005; Renouf et al., 2005).

Several authors have manifested that controlling the growth of spoiler microorganisms is one of the most important challenges of the current winemaking process (Wedral et al., 2010; Capozzi et al., 2016). This issue is critical for aged wines because of their added value. Multiple methods detect spoilage wine bacteria and yeast, but most are based on culture-dependent techniques (Nisiotou and Gibson, 2005; Martorell et al., 2006; Renouf et al., 2007; Guzzon et al., 2017; Izquierdo-Cañas et al., 2018). These techniques have been proven to be biased and not effective to detect the viable but non-culturable (VBNC) microorganisms, which are presumably abundant during aging due to challenging conditions for most microorganisms (high alcohol, low nutrients, high acidity, anaerobiosis, SO_2_) (Serpaggi et al., 2012). This resistance phenomenon may be reversed when the environmental parameters change (SO_2_, pH, and O_2_) and trigger additional fermentation start during barrel maturation or bottle-aging (Capozzi et al., 2016). The metabolic activities of microorganisms at these stages might be detrimental to the wine flavor (Renouf et al., 2005). The introduction of molecular methods based on DNA has improved the detection of present cells even at low concentrations. However, most of the studies dealing with microbiological spoilage of wine are focused on the detection of a few specific species that had been previously associated with such deterioration (Hierro et al., 2006; Andorrà et al., 2010a; Tofalo et al., 2012). Thus, the use of high-throughput sequencing (HTS) techniques could provide a more realistic view of the complex microbial community present during wine aging. These techniques have been recently used in wine samples mostly focusing on grapes, grape must, or fermentation stages (Bokulich et al., 2014, 2015, 2016; Piao et al., 2015; Pinto et al., 2015; Marzano et al., 2016; Salvetti et al., 2016; Lleixà et al., 2018; Mezzasalma et al., 2018; Gao et al., 2019; Sirén et al., 2019). However, little attention has been paid to changes of microbial communities during wine aging process or factors driving its evolution. It is well recognized that factors affecting wine composition are grape variety, aging time, wood origin, along with its toasting level during barrel aging (Spillman et al., 1997; Garde-Cerdán and Ancín-Azpilicueta, 2006; Cadahía et al., 2009; Gómez-Plaza and Cano-López, 2011) and SO_2_ addition or the stopper composition during bottle aging (Arapitsas et al., 2014, 2018; Azevedo et al., 2014). Nevertheless, the influence of these factors over the present microbial communities during aging is not known.

In the present study, red wines were analyzed during aging to monitor the taxonomic composition of prokaryotic and eukaryotic communities by HTS of short amplicons of hypervariable domains of 16S rDNA gene and ITS1–ITS2. The aging process of wines was performed in the only two Spanish Qualified Appellations of Origin: DOQ Priorat (Catalonia) and DOCa Rioja (Spain) regions. The factors considered for the comparison included time of wine aging in the barrels, prior usage of the barrels, and, in the case of Rioja wines, differences between wine aging in oak barrels or glass bottles.

## Materials and Methods

### Samples

**FIGURE 1:**
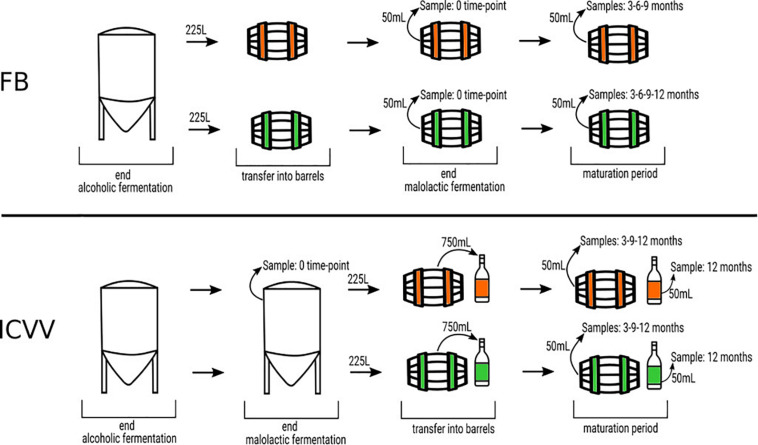
Schematic representation of the experimental setup. The green color represents new barrels and the orange color denotes the used ones, at FB and ICVV cellars. Barrels of 225 L were sampled at different time points, expressed in months, taking 50 ml of wine. In FB, the malolactic fermentation was performed inside the steel tank, whereas in ICVV, it was performed inside the barrels. In addition, in ICVV, the moment the wine was introduced in the barrels, a sample of 750 ml was taken and placed into glass bottles. The bottled wines were sampled after 12 months of maturation at cellar conditions.

The barrel source of red wine samples was the traditional “bordelaise” barrel of 225L and made of French oak, mid-toasted. Two of them are located in a winery of the DOQ Priorat (cellar Ferrer Bobet, FB), and the other two are in the DOCa Rioja (Bodega Institucional, Instituto de Ciencias de la Vid y el Vino, ICVV). Besides the high price of the HTS analysis, we have to consider that the sampled barrels had an increased probability of contamination or oxygenation due to sampling and that is the main reason to keep the number of barrels and the sampled volume low. The procedure of sampling and experimental setup is represented in Figure 1. In each region, the two barrels differed in time of usage, with one barrel being new, without any prior usage (BAN), while the other had been used for 1 year and is referred to as old (BAO). Cleaning of the used barrels were done with the standard cellar practices (washing with pressurized hot water and rinsing). The main parameters of the wine before being introduced in the barrels were similar: 13.8 and 14.1% ethanol; pH 3.3 and 3.4; 0.29 and 0.34 g/L acetic acid; 4.4 and 4.3 g/L tartaric acid (total acidity); 80 and 90 ppm total SO_2_; 1.5 and 1.2 g/L residual sugar; 0.88 and 0.94 g/L malic acid at the end of malolactic fermentation for FB and ICVV, respectively. The barrels followed the habitual cellar management and were maintained with the rest of the barrels from the same vintage. In FB, grape variety was Carignan, which is the main and characteristic variety in DOQ Priorat, and the wine samples were collected at the end of malolactic fermentation inoculated with an autochthonous strain of *Oenococcus oeni*, completed inside BAO and BAN and denoted as 0 time point, at the time points of 3, 6, and 9 months of barrel aging from both barrels, and at the 12-month time point from BAN only, as BAO was accidentally used to refill other barrels due to common practices in the cellar. On the other hand, the grape variety at ICVV winery was Tempranillo, which is the main variety in DOCa Rioja, and the wine samples were collected at the end of spontaneous malolactic fermentation, completed inside the steel tank and denoted as FML or 0 time point, and after 3, 9, and 12 months of barrel aging from both barrels. Additionally, the same day that the wine finished the FML at ICVV winery and transferred into BAO and BAN, a sample of 750 ml from each barrel was taken and bottled into a dark glass bottled as the cellar uses for its wine commercialization. These bottle-aged wine samples, from the old (BTO) and new (BTN) barrel, were stored in the same cellar as the barrels and analyzed after 12 months of bottle aging. At each sampling point, we sampled three bottles of 50 ml of aged wines with a sterilized pipette of 100 ml introduced into the barrel by a top overture and used for stirring the wine in the barrel and sampling. At the laboratory, we used one of the sample bottles and kept the others at −80°C. From the 50 ml of one bottle, we used 3–10 ml for plating on the different culture media (described at section “Plate Culture”), and 40 ml was filtered through a 0.2-μm polycarbonate filter. The filter was frozen at −80°C and used to extract the DNA once all the samples had been collected in order to avoid differences due to DNA extraction. All the acronyms used for the samples and their descriptions are in Table 1.

**TABLE 1:** Description of the acronyms used for the samples of this study.

**Abbreviation**	**Description**
BAO	Barrel with 1-year prior usage.
BAN	Barrel with no prior usage.
BTO	1-year wine sample that was sampled from BAO barrel and aged in bottles.
BTN	1-year wine sample that was sampled from BAN barrel and aged in bottles.
FML_0	Malolactic fermentation completed inside steel tank.
BAO_0	Malolactic fermentation completed inside BAO.
BAO_3	3-month wine sample aged in BAO.
BAO_6	6-month wine sample aged in BAO.
BAO_9	9-month wine sample aged in BAO.
BAO_12	12-month wine sample aged in BAO.
BAN_0	Malolactic fermentation completed inside BAN.
BAN_3	3-month wine sample aged in BAN.
BAN_6	6-month wine sample aged in BAN.
BAN_9	9-month wine sample aged in BAN.
BAN_12	12-month wine sample aged in BAN.

### Sequencing Library Construction

The library construction included the DNA extraction protocol that follows the recommended procedure of the DNeasy Plant Mini kit (Qiagen, Hilden, Germany), including three bead-beating steps for 3 min in a FastPrep-24 bead beater (MP Bio, Solon, OH, United States) (Lleixà et al., 2018). Extracted DNA quantity was checked by nanodrop and sent to CRG (Centre for Genomic Regulation, Barcelona, Spain). The DNA quality was checked by the Agilent 2100 Bioanalyzer, and the quantity was adjusted to 10 μg per sample in order to be sequenced by Illumina MiSeq 2x300, using the primers 341F/785R for the 16S amplicon (Herlemann et al., 2011) and ITS2F/ITS2R for the ITS amplicon (White et al., 1990).

### Bioinformatic and Statistical Analysis

The processing of the raw amplicon sequences has been performed using Quantitative Insights into Microbial Ecology (QIIME versions 1.9.1 and 2018.2) implementing the Illumina OTU pipeline steps previously described (Kioroglou et al., 2019) with a Phred33 quality filtering threshold of <20, 99% similarity threshold during OTU clustering, and BLAST+ as taxonomic classification algorithm (Camacho et al., 2009). After quality filtering and taxonomic classification, exclusion of sequences matching chloroplast or mitochondria was performed. The sequences obtained during this study have been included in the SRA database of the NCBI under the BioProject accession number PRJNA635684.

Moreover, due to the nature of the OTU counts data, such as sparsity and lack of normality, as well as the compositionality constraint applied after converting the OTU counts to relative abundancies, non-parametric methodologies are necessary for the statistical analysis of the resulted OTU counts that do not depend on relative abundancies and assumptions (Tsilimigras and Fodor, 2016; Weiss et al., 2017). Therefore, in the current study, we have implemented the compositional analysis toolbox GNEISS (Morton et al., 2017), as incorporated into Quantitative Insights Into Microbial Ecology framework (QIIME version 2019.1) (Bolyen et al., 2018).

Statistical analysis has been based on the factors barrel type and time. For FB, the factor barrel-type included the 0-, 3-, 6-, and 9-month barrel-aged wine samples separated in the groups of old and new barrel resulting in four samples per group, whereas the factor time concerned the barrel-aged wine from old and new barrel grouped by the attributes 0-, 3-, 6-, and 9-month time points leading to two samples per group. Similarly, for ICVV, the factor barrel type concerned the 3-, 9-, and 12-month barrel-aged wine samples divided into the groups of old and new barrel, and the factor time comprised the four groups of 3-, 9-, and 12-month barrel-aged, and 12-month bottle-aged 1 wine samples. Moreover, ICVV included the additional factor bottled wine which included the 12-month barrel-aged and 12-month bottle-aged wine samples. Summarizing, for the statistical analysis, the samples were distributed as four of old barrel compared to four of new barrels (at each cellar), two samples for each of the four time points (at each cellar), and two samples for glass bottle compared with barrel samples (just for ICVV cellar) (Table 1). Using the rarefied OTU table, alpha diversity was calculated based on the Shannon index, and statistical significance at alpha level 0.05 was evaluated using Student’s *t*-test for the factor barrel type and ANOVA for the factor time. The rarefied OTU table also became the source for assessing the beta diversity that was based on the Bray–Curtis index since taxonomy was constrained at genus level. The resulting Bray–Curtis distance matrix became the input for principal coordinate analysis (PCoA) as well as permutational multivariate analysis of variance (PERMANOVA) using alpha level 0.05 and the factors barrel type and time. On the other hand, GNEISS utilized the unrarefied OTU table since it applies its own normalization. In a nutshell, GNEISS performed zero OTU counts imputation and clustering of the genera into two groups via Ward hierarchical clustering. Upon these two groups, which are considered anti-correlated, GNEISS applied isometric log-ratio transformation with one group being the numerator and the other the denominator. Therefore, this transformation provides a log ratio, referred to as balance, which may have a positive or negative value and reflects for a given sample the changes that might have occurred in the OTU counts of the genera from the numerator, the denominator, or both in relation to another sample. Finally, the average impact of each one of the identified genera on the balances has been calculated by the following equation:

$$I\,m\,p\,a\,c\,t_{\text{i}}=\frac{1}{n}\,\sum_{j=1}^{n}2^{\left|B_{j}-b_{j}\right|}$$

where *n* refers to the total number of samples, *B*_*j*_ refers to the log2 ratio of all OTU counts of the genera included in the numerator and denominator, and *b*_*j*_ refers to the log2 ratio for the *j*th sample after subtracting the OTU counts of the *i*th genus belonging to either the numerator or the denominator. Therefore, this impact represents the average fold change caused on the log2 ratio from a given genus. The greater the impact, the greater the influence of this genus on the balances calculated by GNEISS.

### qPCR Analysis

Quantitative PCR (qPCR) was performed on the extracted DNA to quantify the main microorganisms previously detected in wine according to Lleixà et al. (2018). The used primers anneal the ribosomal gene region and allowed the quantification of total yeasts, *Saccharomyces* genus, *Hanseniaspora* genus, *Starmerella bacillaris*, *Botrytis cinerea*, acetic acid bacteria (AAB), and lactic acid bacteria (Lleixà et al., 2018) and DBRUXF/DBRUXR for *Brettanomyces bruxellensis* (Phister and Mills, 2003). Standard curves were created by plotting the *C*t (cycle threshold) values of the qPCR performed on dilution series of cells against the log input cells/ml. Samples and cultures for standard curves were analyzed in triplicate.

### Plate Culture

Samples were serially diluted in sterile water and plated on (i) YPD medium (glucose 2%, peptone 2%, yeast extract 1%, agar 1.7%) and incubated at 28°C for 48 h; (ii) modified WLN medium (Difco^TM^ WL Nutrient Medium, BD) with the addition of cycloheximide to suppress yeast growth (100 mg/L) and incubated from 7 to 10 days at 28°C; (iii) MRS Agar medium (De Man et al., 1960) supplemented with 4 g/L L-malic acid, 5 g/L fructose, 0.5 g/L L-cysteine, 100 mg/L nystatin, and 25 mg/L sodium azide adjusted to pH 5.0 and incubated at 28°C in a 10% CO_2_ atmosphere; (iv) GYC-Ca Agar medium (glucose 5%, yeast extract 1%, CaCO_3_, and agar 2%, pH 6.3) supplemented with 100 mg/L natamycin to suppress yeast growth and incubated at 28°C for 3–5 days under aerobic conditions. Appropriate dilution plates were counted. The YPD medium provided the total yeast counts, modified WLN medium is selective for *Brettanomyces* genus, whereas MRS medium and GYC-Ca provided LAB and AAB counts, respectively.

## Results

### Sequence Analysis and Alpha Diversity

**FIGURE 2:**
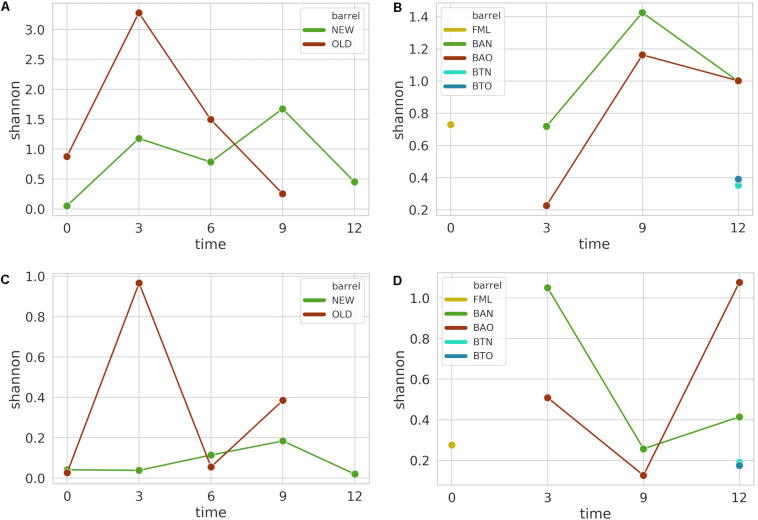
Alpha diversity is based on Shannon index for FB 16S **(A)** and ITS **(C)** amplicon samples, as well as for ICVV 16S **(B)** and ITS **(D)** amplicon samples. Acronyms BAN or NEW and BAO or OLD refer to barreled wine from new and old barrel, respectively; FML refers to final malolactic fermentation stage; and BTN and BTO refer to bottled wine from new and old barrel, respectively.

From the initial 1,066,085 16S amplicon raw sequences for FB and 1,520,976 for ICVV, a corresponding sequence filtering of 11 and 5% resulted after applying all the filtering steps, leading to a rarefication threshold of 30,815 sequences per sample for FB and 101,243 sequences per sample for ICVV. The rarefaction curves of 16S and ITS sequences are included in the Supplementary Figure S1. Shannon alpha diversity did not reveal statistical significance for FB (Figure 2A) for any of the factors, whereas statistical significance (*p*-value 0.02) for the factor time was observed for ICVV’s samples (Figure 2B). Overall, the barrel-aged wine from old and new barrel exhibited similar trends in both cellars, whereas the 12th month bottle-aged wine resulted in lower diversity than the 12th month barrel-aged wine in the case of ICVV.

For the ITS amplicon, the initial 1,252,877 raw sequences of FB were filtered by 38% and by 29% the initial 1,366,522 raw sequences of ICVV, resulting in a rarefication threshold of 2832 sequences per sample for FB and 2381 sequences per sample for ICVV. Shannon alpha diversity was found to have statistically non-significant differences between the groups of the factors barrel type and time for FB (Figure 2C) and ICVV (Figure 2D) with the bottle-aged wine having once again lower diversity than the barrel-aged wine.

### Principal Coordinate Analysis of Samples

**FIGURE 3:**
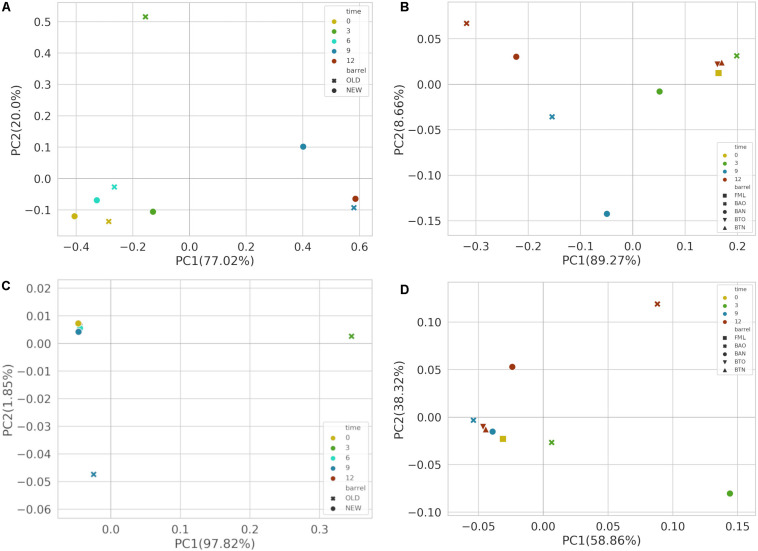
Principal coordinate analysis (PCoA) based on Bray–Curtis distance metric for FB 16S **(A)** and ITS **(C)** amplicon samples, as well as for ICVV 16S **(B)** and ITS **(D)** amplicon samples. Acronyms BAN or NEW and BAO or OLD refer to barreled wine from new and old barrel, respectively; FML refers to final malolactic fermentation stage; and BTN and BTO refer to bottled wine from new and old barrel, respectively.

After performing PCoA based on Bray–Curtis distance metric, bacterial communities presented a separation between early (<6) and late (>9) maturation wine samples across the first principal component for FB (Figure 3A) accompanied by statistical significance (*p*-value 0.05) for the factor time. Similar clustering of bacterial communities became apparent also across the first principal component between the early (<9) and late (>9) maturation wine samples of ICVV (Figure 3B) with a reported statistical significance (*p*-value = 0.03) for the factor time. Along the same component, a clear separation between the barrel and bottle-aged wine bacterial communities could also be observed.

For FB, PCoA analysis based on Bray–Curtis of the fungal communities (Figure 3C) presented separation of the 3- and 9-month BAO samples from the rest; however, PERMANOVA reported non-significant differences between the groups of factors barrel-type and time. On the other hand, for the fungal communities of ICVV samples (Figure 3D), the reported statistical significance based on the factor time (*p*-value = 0.03) seems to refer to the 3-month BAN and 12-month BAO samples due to their greater distance to the rest of the samples. Regarding the factor bottled wine, a higher degree of separation could be observed between the fungal communities of BTO and BAO samples than between BTN and BAN samples.

### Influence of Studied Factors on Bacterial Communities

**FIGURE 4:**
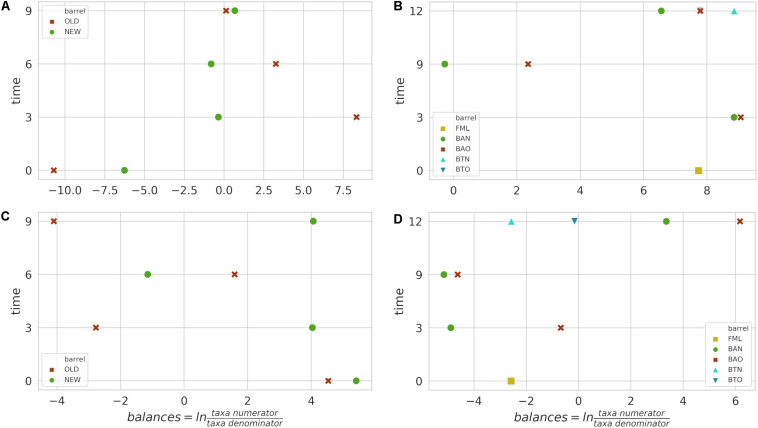
Calculated balances by GNEISS for FB 16S **(A)** and ITS **(C)** amplicon samples, as well as for ICVV 16S **(B)** and ITS **(D)** amplicon samples. Acronyms BAN or NEW and BAO or OLD refer to barreled wine from new and old barrel, respectively; FML refers to final malolactic fermentation stage; and BTN and BTO refer to bottled wine from new and old barrel, respectively.

**FIGURE 5:**
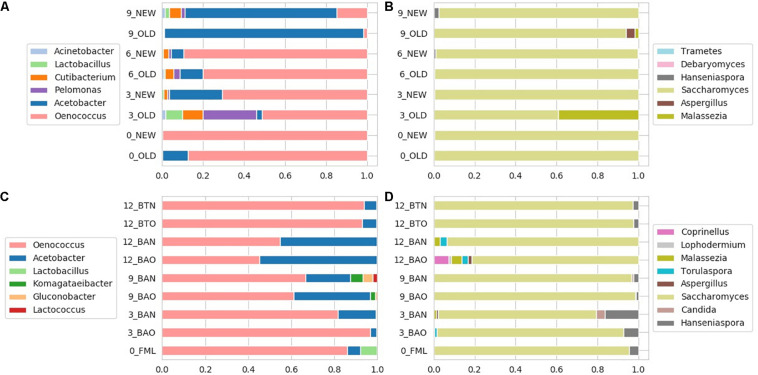
Relative abundances for FB 16S **(A)** and ITS **(B)** amplicon samples, as well as for ICVV 16S **(C)** and ITS **(D)** amplicon samples. Acronyms BAN or NEW and BAO or OLD refer to barreled wine from new and old barrel, respectively; FML refers to final malolactic fermentation stage; and BTN and BTO refer to bottled wine from new and old barrel, respectively.

**FIGURE 6:**
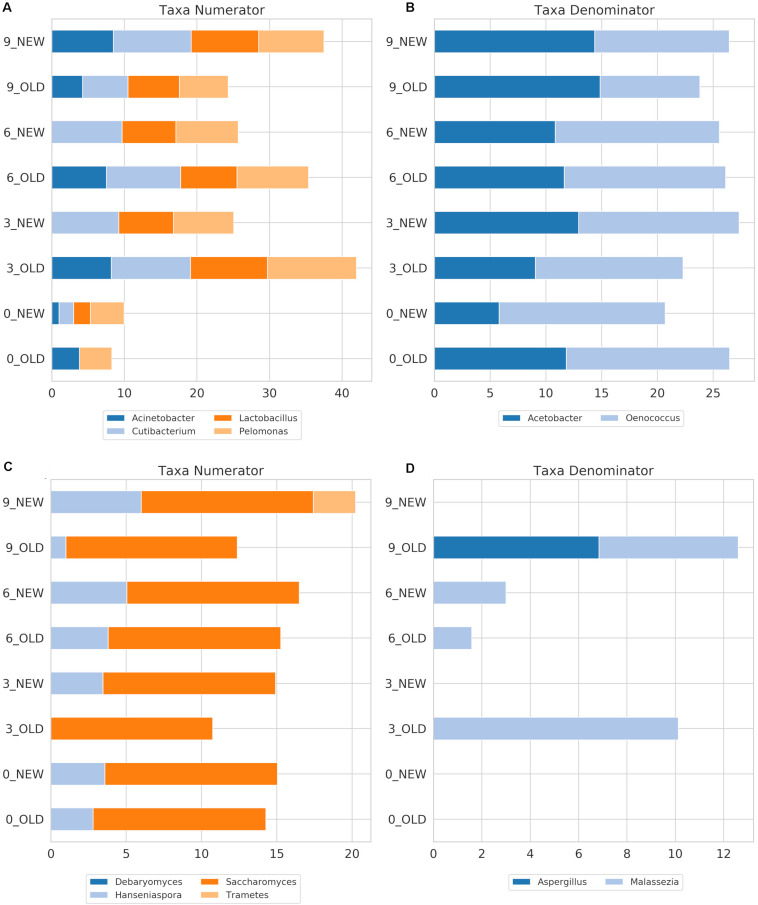
FB log2-transformed OTU counts of the genera with the highest impact on the balances for the amplicons 16S **(A,B)** and ITS **(C,D)** that correspond to the numerator or the denominator of the balances. The indicators NEW and OLD refer to the new and old barrel, respectively, and numbers at the beginning of the indicators refer to the sampling period.

The identified bacterial genera for FB along with their rarefied OTU counts are given in Table 2, and the calculated balances by GNEISS based on these bacterial genera are provided in Figure 4A. Overall, the balances did not show statistically significant differences between the groups of the factors barrel type and time, and the genera that seem to have greatly influenced the balances are *Acinetobacter*, *Cutibacterium*, *Lactobacillus*, *Pelomonas*, *Acetobacter*, and *Oenococcus.* In Figure 5A, the relative abundances of these genera are given, whereas in Figures 6A,B, their log2-transformed OTU counts are shown.

**TABLE 2:** Rarefied OTU counts for FB (Priorat) cellar 16S taxonomy.

**Taxonomy**	**FB_BAO_0**	**FB_BAO_3**	**FB_BAO_6**	**FB_BAO_9**	**FB_BAN_0**	**FB_BAN_3**	**FB_BAN_6**	**FB_BAN_9**	**FB_BAN_12**
*Leuconostoc*^a^	0	1	0	0	0	27	0	0	0
*Bradyrhizobium*^a^	0	604	0	1	1	0	52	0	0
*Lactobacillus*^a^	0	1570	221	134	4	187	169	633	276
*Corynebacterium*^*a*^	0	926	0	0	0	1	0	0	0
*Delftia*^a^	3	1576	142	0	0	25	219	741	0
*Staphylococcus*^a^	4	622	146	28	1	58	0	257	109
*Streptococcus*^a^	0	0	134	0	0	100	233	501	145
*Deinococcus*^a^	0	0	1518	1	0	0	0	0	0
*Stenotrophomonas*^a^	2	1547	155	2	0	0	0	72	0
*Pelomonas*^a^	21	4929	917	106	24	313	387	510	274
*Acinetobacter*^a^	13	295	184	18	1	0	0	359	477
*Thermicanus*^a^	0	863	0	0	0	0	0	0	0
*Rothia*^a^	0	398	0	0	0	56	0	1	0
*Cloacibacterium*^a^	0	3253	0	0	0	0	0	0	0
*Vulcaniibacterium*^a^	0	0	197	19	0	0	0	0	0
*Pseudomonas*^a^	4	1667	0	25	0	32	0	229	57
*Bacteroides*^a^	0	13	0	0	0	0	0	0	0
*Cutibacterium*^a^	0	1919	1183	75	3	594	824	1679	230
*Glutamicibacter*^a^	0	400	3	0	0	0	0	3	0
*Oenococcus*^b^	25,553	9697	22,808	493	30,679	21,541	27,101	4305	20
*Acidovorax*^b^	1032	3	0	0	0	0	0	1	1
*Aquabacterium*^b^	474	1	0	0	0	0	0	138	0
*Komagataeibacter*^b^	0	0	0	0	9	0	0	0	0
*Lonsdalea*^b^	2	0	0	0	0	0	0	0	0
*Acetobacter*^b^	3669	531	3207	29,913	55	7870	1830	21,385	29,158
*Candidatus Finniella*^b^	3	0	0	0	0	0	0	0	0
*Gluconobacter*^b^	0	0	0	0	21	11	0	1	0
*Flavobacterium*^b^	35	0	0	0	17	0	0	0	68

*Genera included in the numerator of the calculated balances by GNEISS are denoted with (a), whereas those included in the denominator are denoted with (b). BAN, barrel new; BAO, barrel old.*

The most abundant bacterial genus at FB was *Oenococcus*, which had an average of over 80% of the bacterial sequences the first 6 months of maturation, followed by *Acetobacter*, which increased in abundance at the end of barrel maturation representing above 85% of the sequences at the 9th month samples (Figure 5A).

**FIGURE 7:**
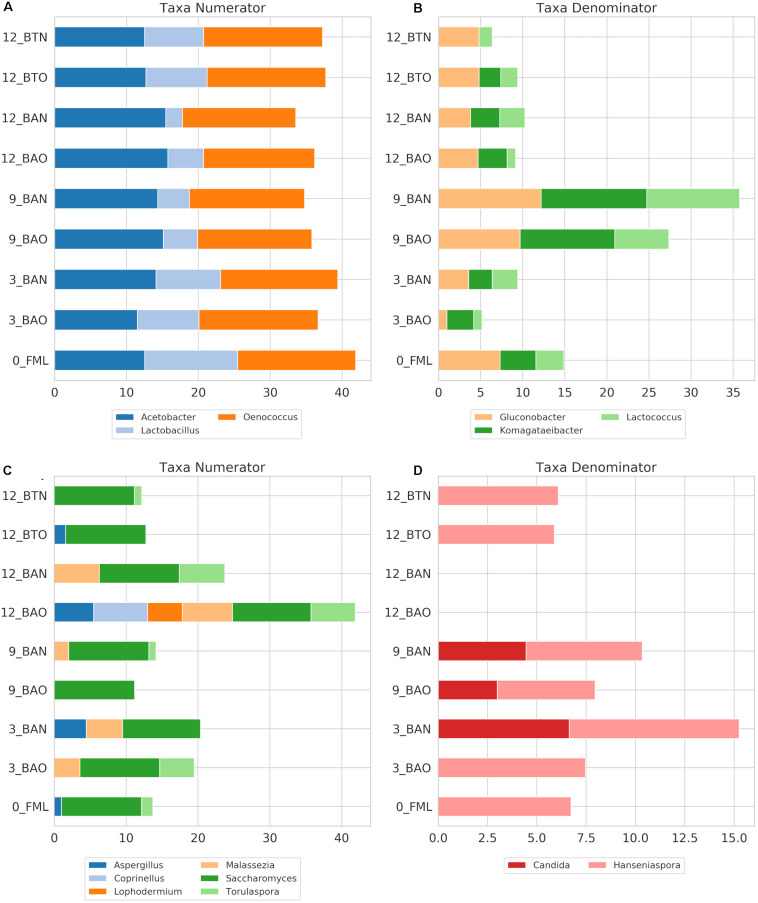
ICVV log2-transformed OTU counts of the genera with the highest impact on the balances for the amplicons 16S **(A,B)** and ITS **(C,D)** that correspond to the numerator or the denominator of the balances. Acronyms BAN and BAO refer to barreled wine from new and old barrel, respectively; FML refers to final malolactic fermentation stage; and BTN and BTO refer to bottled wine from new and old barrel, respectively. Numbers at the beginning of the acronyms refer to the sampling period.

Regarding ICVV samples, Table 3 holds the rarefied OTU counts of the identified bacterial genera, Figure 4B provides their calculated balances, and their relative abundance is displayed in Figure 5C. Similarly to FB, the most abundant bacterial genus was *Oenococcus*, that had an average of relative abundance above 60% the first 9 months of maturation. However, even though *Acetobacter* increased at the end of barrel maturation, its abundance was slightly lower than that in FB, representing 30 and 50% of the bacterial sequences at the 9th and 12th months of barrel maturation (Figure 5C). Interestingly, the relative abundances of the detected bacterial genera did not change after 12 months of glass bottle maturation (Figure 5C). The balances revealed statistically significant differences between the groups of the factor time with *Acetobacter*, *Oenococcus*, *Lactobacillus*, *Gluconobacter*, *Lactococcus*, and *Komagataeibacter* being the main genera that drove these differences (Figures 7A,B). In both cellars, *Acetobacter* and *Oenococcus* have been included in the same group by GNEISS as they have been identified of being correlated. Acetobacter exhibited an increasing trend through time in both cellars, whereas the abundance of *Oenococcus* was relatively stable in ICVV but decreased over time in the case of FB. That could be explained by the fact that the initial wine samples of FB were taken at the end of malolactic fermentation where the abundance of *Oenococcus* was at higher levels. On the other hand, *Lactobacillus* displayed different behavior between the two cellars, with an increasing tendency in the case of FB and a decreasing one in the case of ICVV. Overall, the barrel-aged wine did not present great differences between the old and new barrels. The observed differences between the 12th month bottle and barrel-aged wine could be attributed to *Acetobacter* and *Lactobacillus* whose abundances in the bottle-aged wine were similar to those of the early maturation period (<9).

**TABLE 3:** Rarefied OTU counts for ICVV (Rioja) cellar 16S taxonomy.

**Taxonomy**	**Rioja_FML0**	**Rioja_BAN3**	**Rioja_BAN9**	**Rioja_BAN12**	**Rioja_BAO3**	**Rioja_BAO9**	**Rioja_BAO12**	**Rioja_BTN12**	**Rioja_BTO12**
*Oenococcus*^a^	87,265	82,877	67,457	55,563	97,918	61,883	45,720	95,026	94,129
*Acetobacter*^a^	5996	17,848	21,020	45,646	2935	35,985	55,457	5893	6715
*Lactobacillus*^a^	7796	494	20	4	380	26	30	295	364
*Komagataeibacter*^b^	18	6	6000	10	8	2443	10	0	5
*Gluconobacter*^b^	159	11	4653	13	1	821	25	27	27
*Lactococcus*^b^	9	7	2093	7	1	85	1	2	3

*Genera included in the numerator of the calculated balances by GNEISS are denoted with (a), whereas those included in the denominator are denoted with (b). FML, final malolactic fermentation; BAN, barrel new; BAO, barrel old; BTN, bottle new; BTO, bottle old.*

The analysis of qPCR showed a deep decrease with time of LAB after the introduction of the wine in barrels, but the number of AAB remained constant with time in the barrels and also in the glass bottles (Table 4). The cfu of LAB on plates were just one order below the cells detected by qPCR. However, no cells were recovered on the medium for AAB (Table 4).

**TABLE 4:** Mean values of qPCR (cells/ml) and plate culture count (cfu) analysis on the studied samples from FB **(A)** and ICVV **(B)**.

**(A)**										
		**FB**								
		**BAO 0**	**BAO 3**	**BAO 6**	**BAO 9**	**BAN 0**	**BAN 3**	**BAN 6**	**BAN 9**	**BAN 12**
qPCR	Total yeast	2.95E+06	5.46E+03	4.12E+05	1.98E+04	2.14E+06	6.71E+05	2.81E+05	2.05E+04	4.06E+04
	*S. cerevisiae*	1.22E+06	1.67E+03	1.51E+05	1.95E+03	6.77E+05	3.16E+05	1.95E+03	5.41E+03	2.29E+04
	*S. bacillaris*	2.28E+04	−	−	−	2.44E+04	−	−	−	−
	*H. uvarum*	2.71E+04	−	−	−	2.66E+04	1.23E+04	−	−	−
	*B. bruxellensis*	−	−	−	6.80E+01	−	−	2.70E+01	−	−
	LAB	1.27E+06	−	−	−	2.08E+06	1.59E+04	−	−	−
	AAB	5.31E+06	−	−	2.06E+06	2.06E+06	8.76E+05	2.06E+06	1.85E+05	8.62E+05
Plate count	YPD (Yeasts)	1.01E+03	2.00E+02	1.24E+03	5.00E+01	4.25E+02	9.00E+01	1.70E+02	7.35E+02	5.70E+02
	WLN (*Brettanomyces*)	−	−	−	9.50E+01	−	−	−	−	−
	MRS (LAB)	1.67E+05	1.30E+03	−	−	1.13E+05	−	−	−	−
GYC-Ca (AAB)	−	−	−	−	−	−	−	−	−	

**(B)**										
		**ICVV**								
		**FML 0**	**BAO 3**	**BAO 9**	**BAO 12**	**BAN 3**	**BAN 9**	**BAN 12**	**BTO 12**	**BTN 12**

qPCR	Total yeast	2.34E+06	4.76E+03	6.34E+04	3.39E+03	6.80E+05	1.97E+05	1.16E+04	2.14E+05	9.28E+05
	*S. cerevisiae*	5.97E+05	1.59E+03	2.30E+04	6.58E+02	1.87E+05	5.23E+04	3.74E+03	8.26E+04	2.87E+05
	*S. bacillaris*	4.12E+05	−	−	−	−	−	−	5.39E+04	3.72E+04
	*H. uvarum*	2.14E+04	−	−	−	3.92E+03	1.33E+04	-	2.56E+04	8.10E+04
	*B. bruxellensis*	−	−	2.14E+03	3.06E+02	2.74E+02	5.34E+02	−	2.12E+02	−
	LAB	3.58E+06	1.59E+06	2.08E+05	1.06E+05	4.38E+06	2.41E+05	6.48E+04	6.41E+05	1.69E+06
	AAB	2.98E+06	1.40E+06	4.06E+06	2.80E+06	2.13E+06	2.26E+06	1.85E+06	3.09E+06	3.41E+06
Plate count	YPD (yeasts)	1.30E+03	4.50E+02	3.00E+02	2.50E+02	2.38E+02	7.00E+01	9.50E+01	5.20E+02	3.00E+02
	WLN (*Brettanomyces*)	−	−	−	−	−	−	−	−	−
	MRS (LAB)	6.72E+05	1.20E+04	−	−	3.15E+05	−	−	−	−
	GYC-Ca (AAB)	−	−	−	−	−	−	−	−	−

### Influence of Studied Factors on Fungal Communities

The identified fungal genera are reported in Table 5 for FB and in Table 6 for ICVV. The calculated balances of the fungal detected genera are displayed in Figures 4C,D, and their corresponding relative abundances are displayed in Figures 5B,D, respectively. According to the relative abundances of the detected fungal genera (Figures 5B,D), *Saccharomyces* was the predominant yeast at both cellars with an average of 90% of the sequences. Secondly, *Malassezia* was detected at the FB old barrels just at the 3rd and 9th month samples, whereas *Hanseniaspora* was detected at ICCV the first 9 months of the new and old barrel maturation (6%) and at the 12-month bottle-aged samples (3%). Statistical analysis based on the balances of fungal genera verified the statistically non-significant differences between the groups of barrel type and time for FB and showed statistically significant differences for the factor time in ICVV (*p*-value = 0.03). In both cellars, the most abundant genus was *Saccharomyces* with relatively stable abundance over time, and the genera mainly responsible for the observed differences between the samples were *Malassezia* and *Hanseniaspora* for FB (Figures 6C,D) and *Malassezia*, *Hanseniaspora*, and *Torulaspora* for ICVV (Figures 7C,D). GNEIIS analysis of fungal genera identified *Hanseniaspora* and *Malassezia* as anti-correlated in both cellars. The statistical significance for the factor time in ICVV appears to concern the higher number of fungal genera of the 3- and 12-month old barrel samples in comparison to the rest (Figures 7C,D). Finally, once again, the fungal communities of the 12-month bottle-aged wine were different from that of the barrel-aged wine with the BTN and BTO samples having similar composition to FML samples (Figure 7C).

**TABLE 5:** Rarefied OTU counts for FB (Priorat) cellar ITS taxonomy.

**Taxonomy**	**FB_BAO_0**	**FB_BAO_3**	**FB_BAO_6**	**FB_BAO_9**	**FB_BAN_0**	**FB_BAN_3**	**FB_BAN_6**	**FB_BAN_9**	**FB_BAN_12**
*Unidentified*^a^	1	0	1	1	1	1	0	2	0
*Trametes*^a^	0	0	0	0	0	0	0	6	0
*Debaryomyces*^a^	0	0	0	0	0	0	0	0	1
*Hanseniaspora*^a^	6	0	13	1	11	10	32	63	0
*Saccharomyces*^a^	2825	1720	2816	2663	2820	2821	2793	2761	2827
*Aspergillus*^b^	0	0	0	114	0	0	0	0	0
*Malassezia*^b^	0	1112	2	53	0	0	7	0	4

*Genera included in the numerator of the calculated balances by GNEISS are denoted with (a), whereas those included in the denominator are denoted with (b). BAN, Barrel New; BAO, Barrel Old.*

**TABLE 6:** Rarefied OTU counts for ICVV (Rioja) cellar ITS taxonomy.

**Taxonomy**	**Rioja_FML0**	**Rioja_BAN3**	**Rioja_BAN9**	**Rioja_BAN12**	**Rioja_BAO3**	**Rioja_BAO9**	**Rioja_BAO12**	**Rioja_BTN12**	**Rioja_BTO12**
*Coprinellus*^a^	0	0	0	0	0	0	178	0	0
*Lophodermium*^a^	0	0	0	0	0	0	28	0	0
*Malassezia*^a^	0	32	3	77	11	0	124	0	0
*Torulaspora*^a^	2	0	1	79	27	0	71	1	0
*Aspergillus*^a^	1	21	0	0	0	0	44	0	2
*Saccharomyces*^a^	2273	1838	2298	2225	2168	2344	1936	2313	2321
*Candida*^b^	0	99	21	0	0	7	0	0	0
*Hanseniaspora*^b^	105	391	58	0	175	30	0	67	58

*Genera included in the numerator of the calculated balances by GNEISS are denoted with (a), whereas those included in the denominator are denoted with (b). FML, final malolactic fermentation; BAN, barrel new; BAO, barrel old; BTN, bottle new; BTO, bottle old.*

Taking into account qPCR results, the number of yeast represented mostly by *S. cerevisiae* tended to decrease with aging two or three orders of magnitude. However, yeast number in the bottles remained constant or decreased just one order (Table 6). The detection by qPCR of yeast species other than *S. cerevisiae* was non-important except in the case of *S. bacillaris* at ICCV wine at the beginning of aging. The number of yeast cells recovered in plate media was three orders of magnitude lower than the number detected by qPCR but also tends to decrease with aging time (Table 6).

## Discussion

Although deterioration of aging wines in barrels or bottles caused by microorganisms consists a real threat for product quality and marketability, our knowledge on the microbial status of those wines is still quite limited. The present work is one of the first studies dealing with the meta-taxonomic characterization of microbial consortia during aging of wines using a HTS approach. To our knowledge, just one previous meta-taxonomic study included samples from aged wine prior to bottling, but it was mainly focused on the vineyard microbiota and its correlation with the chemical composition of the finished wines (Bokulich et al., 2016). That study used HTS at aged wines and detected a high number of bacterial and fungal genera allowing to differentiate wines from different vineyards (Bokulich et al., 2016). In addition, for the first time, our work used a meta-taxonomic approach to monitor the effect of time of aging in the barrels, prior usage of the barrels, and aging in glass bottles over the bacterial and eukaryotic communities.

Diverse studies found that species diversity tends to decrease over time during the winemaking process, the two most significant decrease being during alcoholic fermentation and after SO_2_ addition once fermentation is finished (Renouf et al., 2007). The lower species diversity could be explained by the stressing conditions (high ethanol concentration, low pH, and scarcity of nutrients) that characterize the process resulting in a strong selection. However, through the aging period, the microbial diversity has been shown to be relatively constant, although the number of cells tends to decrease (Andorrà et al., 2010b). Overall, our results showed non-significant changes in microbial diversity during aging for the factors aging time and barrel type. The exception was found for ICVV samples that showed an inflexion point in bacterial and fungal diversity over time at 9 months of aging, harboring the 3- and 12-month samples’ higher diversity. Meanwhile, our qPCR results pointed to a decrease in yeast and LAB numbers through time, whereas the AAB population were kept constant, as previously observed (Andorrà et al., 2010a). However, no AAB cells were recovered by culturing in each stage, manifesting the difficulty to grow them under laboratory conditions or their VBNC state (Torija et al., 2010; Navarro et al., 2013). These results point out that either the death of the cells or their entrance into VBNC state in the barrels led to a decreased cell number but nonetheless a scarce fluctuation of their diversity based on the factors time and barrel type. However, the 12-month bottle-aged wine resulted in significantly lower diversity of bacterial and fungal communities compared to the 12-month barrel-aged wine, while the number of cells was similar or even one order higher than that in the bottles, suggesting that the barrel has a positive influence on the microbial diversity. In fact, the diversity and number of cells of bottled samples were similar to those of the wine samples just introduced in the barrels after FML. Wood is more or less porous depending on the origin of the wood. For example, American and French oak have different porosity, and their absorbent structure allows progressive microbial penetration, especially during the first time it is used (Renouf et al., 2005). These microorganisms can develop when they come into contact with wine, increasing the diversity and with a possibly harmful effect on wine quality.

The relative abundances alone of the detected bacterial and fungal genera give little information about the microbial communities’ changes according to the studied factors. Thus, in this study, we analyzed the calculated balances by GNEISS based on these genera. Even if no profound changes were observed for microbial diversity across aging time in barrels, bacterial communities of early and late maturation differed significantly at both ICVV and FB wines. The bacterial changes between early and late aging are in agreement with the chemical evolution previously observed for the wines during aging (Spillman et al., 1997; Garde-Cerdán and Ancín-Azpilicueta, 2006; Cadahía et al., 2009; Gómez-Plaza and Cano-López, 2011). On the other hand, fungal communities behaved differently at the two cellars but they have in common that the final communities at 12 months of barrel aging were similar to the samples of the initial aging, harboring the intermediate aging samples’ different fungal communities. The HTS and qPCR techniques based on DNA do not allow the differentiation between lives, VBNC, or death cells. However, the combination of both techniques allowed us to know that the number of yeasts was decreasing with time while their structure and diversity were not changing deeply. Also, the cells detected by plate culture were culturable while the rest detected by qPCR but not recovered on plates would be either dead or in the VBNC state. The VBNC state may be reversed when the environmental parameters are adequate and the metabolic activities of recovered microorganisms might be detrimental to the wine flavor (Renouf et al., 2005).

The number of times the barrels are used determines the oak composition and the rate of chemical compounds extracted from the wood (Garde-Cerdán and Ancín-Azpilicueta, 2006). Similarly, it is well known that aroma of wines aged in oak barrels differs significantly from that aged in glass bottles (Aiken and Noble, 1984). Thus, changes in the concentration of different compounds during oak aging due to those factors could potentially affect the population of microorganisms in the samples. In fact, in our study, 12-month barrel- and bottle-aged wines harbored different bacterial communities. This was also the case for fungal communities specially for wine in older barrels. Changes in microbial composition together with the higher diversity observed in the barrel with respect to the glass bottle indicated the possible positive effect of the former on the development of new species even if the total number of yeasts and LAB was decreasing with time. However, the factor barrel type did not significantly influence the bacterial or fungal communities’ composition, probably because just 1 year of barrel usage was not enough to infer deep changes.

Nisiotou and Gibson (2005) were the first to study culturable yeast on bottled wine and the yeast isolates were mainly *B. bruxellensis*, *S. cerevisiae*, and *Rhodotorula pinicola*. Other microorganisms like the bacteria *O. oeni* or *Pediococcus parvulus* or the yeasts *Pichia anomala* or *Zygosaccharomyces bailii* have been previously isolated and detected during wine aging (Renouf et al., 2007). Furthermore, Rubio et al. (2015) found that *Brettanomyces* presence (cfu/ml and strains) and ethylphenol production during aging were affected more by the aging conditions (aerobic/anaerobic and sulfiting) than by the origin of the oak. However, most of the microbiological studies of aging wines have been focused on specific spoilage microorganisms and their effects over wine quality. In our study, the HTS allowed us the detection of high diversity of bacterial and fungal genera in the absence of any sign of wine spoilage or off-odors according to cellar monitoring. Overall, the observed changes in bacterial communities across the different studied factors resulted from changes in the balances of the genera *Acetobacter*, *Oenococcus*, *Lactobacillus*, *Lactococcus*, and *Komagataeibacter*, with the first two being the most abundant at both cellars. In the case of fungal genera, the most abundant was *Saccharomyces*, and together with *Malassezia* and *Hanseniaspora* (and *Torulaspora* for ICVV), we determined the differentiation of the samples at intermediate aging time. The fact that *Hanseniaspora* and *Malassezia* have been identified as anti-correlated in both cellars could suggest an underlying competition between these two genera. *Saccharomyces*, *Hanseniaspora*, and *Torulaspora* are frequently reported genera in wine using either culture or molecular-based techniques (Pinto et al., 2015; Padilla et al., 2016). *Malassezia* though is a genus naturally found on the skin surface; thus, it is possible that this yeast-like fungus contaminated the samples or the DNA during extraction. Nevertheless, this genus has been recently reported in studies of must and wine samples using HTS (Takahashi et al., 2014; Grangeteau et al., 2017). This methodology has detected minor and rare species that are sometimes overlooked with culture-dependent methods and can detect non-culturable cells at the end of fermentation. However, it is still unknown if these microorganisms have wine environments as their natural niche and have a specific role during winemaking or if they are simply contaminants.

Our results showed that two wines from two cellars obtained from different grape varieties and aged under different conditions resulted in a common number of genera, indicating that the microbial community detected could be the normal in the absence of wine deterioration.

However, Bokulich et al. (2016) also used HTS on wine samples after several months of barrel aging and detected more than 95% of bacterial sequences belonging to *Leuconostoc* (same family as *Oenococcus*) and fungal sequences related to *Cladosporium*, *Botrytis*, and *S. cerevisiae*, in that order of abundance and accounting over 80% of the eukaryotic sequences. Herein, further studies using the newest sequencing technologies would be necessary to elucidate the regular microbial communities during wine aging.

## Conclusion

Barrel aging of wine improves its organoleptic characteristics by the physicochemical reactions occurring between wine and wood compounds. The microorganisms are not supposed to interfere or have a relevant role during wine aging unless uncontrolled growth occurs, thus affecting wine quality. A plethora of studies about the presence of contaminant microorganisms and their by-products during both winemaking and barrel aging are available. However, it is still missing a holistic view of the normal microbiota of aged wine and how the different factors and management affect that microbiota. In the present study, we have used HTS of short amplicons to characterize the bacterial and fungal communities of wines aged for 12 months. The aging in barrels did not significantly affect the microbial diversity with time but changed the structure and composition of fungal and bacterial population. Also, the barrels exert a positive effect on the microbial diversity in comparison with the glass bottles, in which the microbial communities were very similar to those of the samples at the beginning of the aging. Finally, 1-year difference in the usage of the barrels was not enough to induce significant changes in the diversity or composition of wine microbiota through aging. Our results showed that wines from different grape varieties and from different cellars aged under different conditions resulted in a similar microbial composition. Nevertheless, more studies would be necessary to know if that microbiota is the standard after barrel aging or if other factors not considered here could influence it.

## Data Availability Statement

The datasets generated for this study can be found in NCBI SRA, NCBI Accession No. PRJNA635684.

## Author Contributions

AM and MP contributed to the experimentation, funding of the study, and writing of manuscript. DK performed the bioinformatic and statistical analysis and contributed to the writing of the manuscript. All authors contributed to the article and approved the submitted version.

## Conflict of Interest

The authors declare that the research was conducted in the absence of any commercial or financial relationships that could be construed as a potential conflict of interest.

## Supplementary Material

FIGURE S1 Rarefaction curves based on Shannon index of 16S **(A,B)** and ITS **(C,D)** amplicons obtained for FB **(A,C)** and ICVV **(B,D)** samples.
