# Supplementary figures and images for: High-Throughput Sequencing Approach to Analyze the Effect of Aging Time and Barrel Usage on the Microbial Community Composition of Red Wines
Source: Front Microbiol. 2020 Sep 9;11:562560. doi: 10.3389/fmicb.2020.562560 (PMC7509142; doi:10.3389/fmicb.2020.562560)

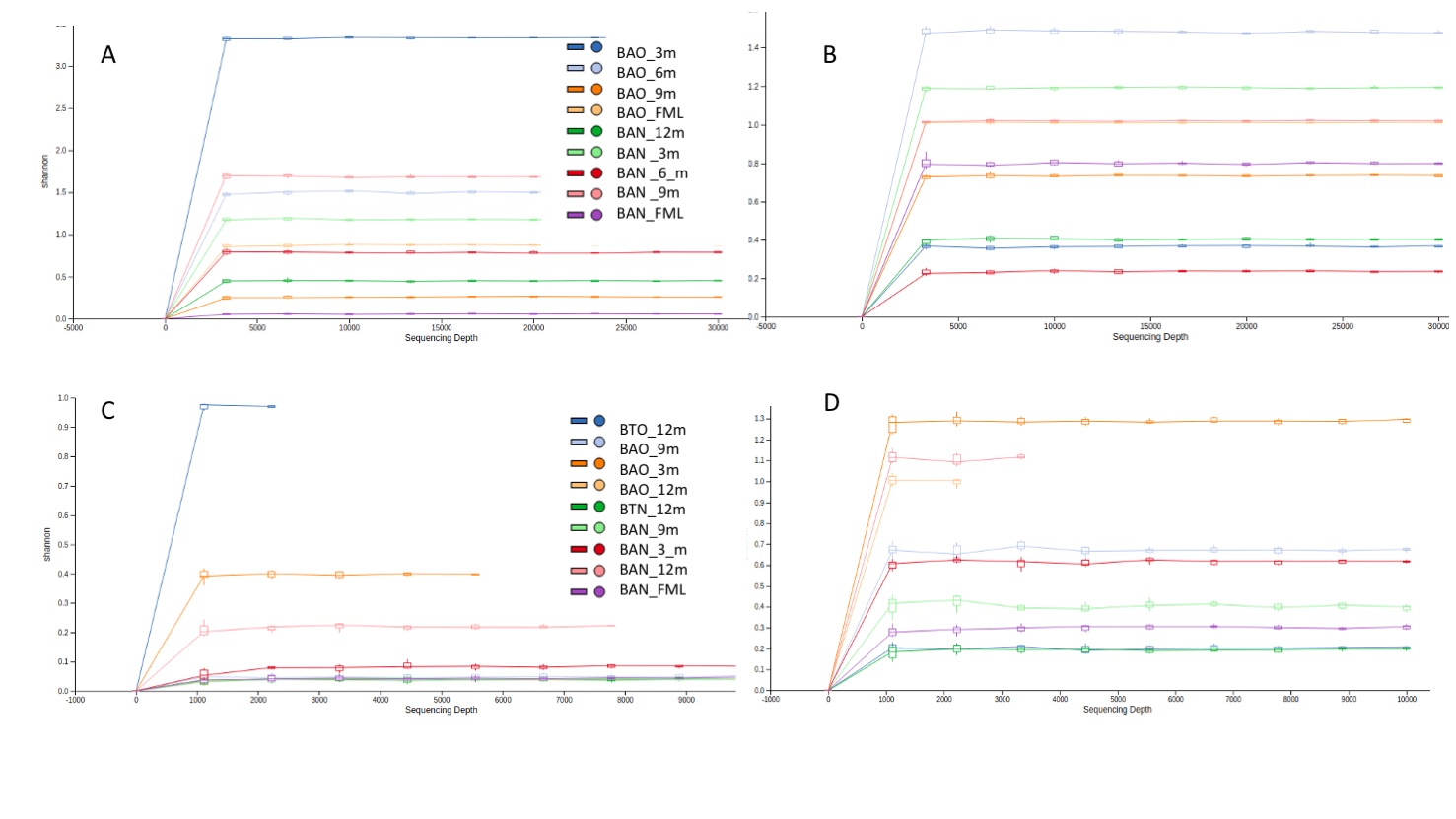

Supplement: FIGURE S1 — Rarefaction curves based on Shannon index of 16S (A,B) and ITS (C,D) amplicons obtained for FB (A,C) and ICVV (B,D) samples. [file Image_1.JPEG]
